# Novel *SLC20A2* variant in a Japanese patient with idiopathic basal ganglia calcification-1 (IBGC1) associated with dopa-responsive parkinsonism

**DOI:** 10.1038/s41439-019-0073-7

**Published:** 2019-09-04

**Authors:** Yaeko Ichikawa, Masaki Tanaka, Eriko Kurita, Masanori Nakajima, Masaki Tanaka, Chizuko Oishi, Jun Goto, Shoji Tsuji, Atsuro Chiba

**Affiliations:** 1 Department of Neurology, Kyorin University School of Medicine, Tokyo, Japan; 2 Department of Neurology, The University of Tokyo Hospital, Tokyo, Japan; 3 Institute of Medical Genomics, International University of Health and Welfare, Chiba, Japan; 4 Department of Neurology, International University of Health and Welfare Mita Hospital, Tokyo, Japan; 5 Department of Molecular Neurology, Graduate School of Medicine, The University of Tokyo, Tokyo, Japan

**Keywords:** Neurological disorders, Medical genetics

## Abstract

Idiopathic basal ganglia calcification-1 (IBGC1) is an autosomal dominant disorder characterized by calcification in the basal ganglia, which can manifest a range of neuropsychiatric symptoms, including parkinsonism. We herein describe a 64-year-old Japanese IBGC1 patient with bilateral basal ganglia calcification carrying a novel *SLC20A2* variant (p.Val322Glufs*92). The patient also presented with dopa-responsive parkinsonism with decreased dopamine transporter (DAT) density in the bilateral striatum and decreased cardiac ^123^I-meta-iodobenzylguanidine uptake.

Idiopathic basal ganglia calcification (IBGC), also known as Fahr disease or primary familial brain calcification (PFBC), is a disorder characterized by bilateral calcifications in the basal ganglia and other brain regions. Clinical manifestations of IBGC range from asymptomatic to neuropsychiatric symptoms, including dystonia, parkinsonism, ataxia, and cognitive impairment^1^. Typically, the inheritance mode of familial IBGC is an autosomal dominant one and to date, four dominant causal genes of familial IBGC have been identified, including *SLC20A2* (IBGC1, MIM: #213600), *PDGFRB* (IBGC4, MIM: #615007), *PDGFB* (IBGC5, MIM: #615483), and *XPR1* (IBGC6, MIM: #616413)^2–5^. Recently, *MYORG* was reported as an autosomal recessive causal gene for IBGC (IBGC7, MIM: #618317)^6,7^. Variants in *SLC20A2*, encoding the type III sodium-dependent phosphate transporter 2 (PiT-2), are a major cause of IBGC^8,9^. Herein, we report an IBGC1 patient with a novel variant in *SLC20A2* associated with dopa-responsive parkinsonism.

The patient was a 63-year-old Japanese woman who presented to our hospital with a one-month history of lumbago and unsteady gait. Neurological examination revealed gait disturbance with stooped posture and short steps, but rigidity, tremor, weakness, and cerebellar symptoms were not observed. Computed tomography (CT) images of her brain revealed marked calcifications in the bilateral basal ganglia, thalami, and dentate nuclei (Fig. 1a). Laboratory tests showed that serum calcium, phosphate, and intact parathyroid hormone levels were all within the normal ranges. There was no family history of IBGC or parkinsonism. After written informed consent was obtained, we analyzed all the coding regions of the IBGC causative genes, *SLC20A2*, *PDGFRB*, and *PDGFB*, by Sanger sequencing as previously reported^10^. We diagnosed her as IBGC1 based on the identification of a novel heterozygous frameshift variant, p.Val322Glufs*92 (NM_006749.4:c.965_966delTG, exon 8), in *SLC20A2* (Fig. 1b). The variant was absent in the following genome databases: dbSNP 151 (https://www.ncbi.nlm.nih.gov/projects/SNP/), Integrative Japanese Genome Variation Database (http://ijgvd.megabank.tohoku.ac.jp/), Exome Aggregation Consortium database version 0.3.1 (http://exac.broadinstitute.org/), and Human Gene Mutation Database (HGMD® Professional 2019.1).

**Fig. 1:**
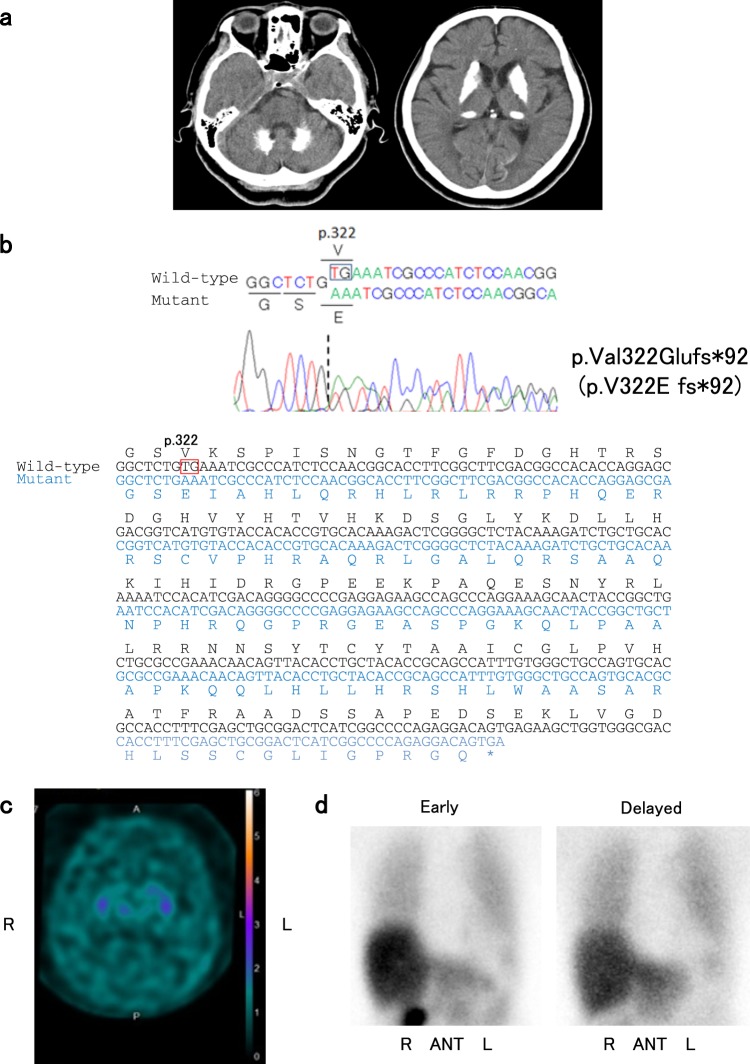
Imaging and sequencing findings of the patient. **a** Computed tomographic (CT) images of the patient show calcifications in the bilateral basal ganglia, thalami, and dentate nuclei. **b** Electropherogram and sequence of *SLC20A2* (NM_006749.4) from the patient’s DNA shows the c.965_966delTG variant. DNA and corresponding amino acid sequences of wild-type and mutant *SLC20A2* alleles are also shown. The c.965_966delTG variant causes a frameshift variant (p.Val322Glufs*92). **c** Dopamine transporter (DAT) single photon emission CT shows diffusely decreased DAT density in the bilateral striatum. The specific binding ratios (SBRs) of both striatum were 0.51 (right) and 0.14 (left). **d** ^123^I-meta-iodobenzylguanidine (^123^I-MIBG) myocardial scintigraphy shows decreased cardiac ^123^I-MIBG uptake with early and delayed heart to mediastinum (H/M) rates of 1.995 and 1.585, respectively

Ten months after her first visit, she was hospitalized because of difficulties in standing up without assistance at the age of 64. She showed severe bradykinesia, postural instability, and mild symmetric rigidity without tremor. Her Unified Parkinson Disease Rating Scale part III (UPDRS-III) score was 43 of 108 on the ninth hospital day. Her Mini-Mental State Examination score was 24 of 30, and her Hasegawa dementia scale revised was 22 of 30. Dopamine transporter (DAT) single photon emission CT using ^123^I-ioflupane showed diffusely decreased DAT density in the bilateral striatum (Fig. 1c). The specific binding ratios (SBRs) of both striatum were 0.51 (right) and 0.14 (left). Her ^123^I-meta-iodobenzylguanidine (^123^I-MIBG) myocardial scintigraphy revealed reduced cardiac ^123^I-MIBG uptake with early and delayed heart to mediastinum (H/M) rates of 1.995 and 1.585, respectively (Fig. 1d). Levodopa therapy (200 mg/day) was started on the 14th hospital day and was effective against bradykinesia and postural instability. She was able to walk without assistance in her room. On the 122nd hospital day, she received 600 mg/day of levodopa, and her UPDRS-III score markedly improved from 43 to 11.

The variants associated with IBGC are located widely in *SLC20A2* among the patients with IBGC, and the correlation of genotype and phenotype remains unclear^1,9,11^.

Parkinsonism is one of the common clinical symptoms of IBGC. Tadic et al. showed that 13% of patients with *SLC20A2* or *PDGFRB* variants presented with parkinsonism^1^. Another review reported motor improvement with dopatherapy in five patients with genetically confirmed IBGC^12^. Genetically confirmed Japanese IBGC1 patients presenting with parkinsonism have also been reported (Table 1)^10,13,14^. Among the five variants summarized in Table 1, two variants (c.516+1G>A and c.965_966delTG) are frameshift variants, presumably resulting in loss of function of SLC20A2. In addition, a decreased level of SLC20A2 protein was described in the case with the missense variant (c.1909A>C, S637R), raising the possibility of unstable mutant protein^13^. Although the functional investigations were not reported for the two missense variants (R71H and G90V), loss-of-function variants are considered for the three variants shown in Table 1. Consistent with previous reports, the majority of variants associated with IBGC are loss-of-function variants^8,9^, and the present study also suggests that loss-of-function mechanisms are likely involved in at least of the three variants. The present case demonstrated decreased DAT density in the bilateral striatum and decreased cardiac ^123^I-MIBG uptake (Fig. 1c, d). The decreased DAT density in the bilateral striatum suggested presynaptic dopaminergic dysfunction, which was reported in patients with IBGC^14–17^. Saito et al. also showed that postsynaptic dopaminergic dysfunction in the bilateral striatum matched calcified regions^16^. These findings suggested that basal ganglia calcification might result in dopaminergic dysfunction in IBGC patients. The three cases with reduced DAT density in the striatum (cases 2, 5, and 7. Table 1) also presented with decreased cardiac ^123^I-MIBG uptake, which was indistinguishable from that observed in patients with Lewy body diseases, including idiopathic Parkinson disease (PD)^18^. Since PD is a relatively common disease in Japan (prevalence of ~150 per 100,000 persons in Japan)^19^, the coincidental presence of idiopathic PD and IBGC remains a possibility concerning dopa-responsive parkinsonism of patients with IBGC1. However, it is important to pay attention to patients with IBGC who show dopa-responsive parkinsonism to provide appropriate treatment. To clarify the etiologies of dopa-responsive parkinsonism occasionally observed in patients with IBGC, further functional analyses including DAT SPECT and ^123^I-MIBG myocardial scintigraphy will be required in a larger number of patients with genetically confirmed IBGC.

**Table 1:** Variants of *SLC20A2* and clinical features of genetically confirmed IBGC1 Japanese patients with parkinsonism

Case	1	2	3	4	5	6	7	8	9
Variant	c.212G>A R71H Exon 2	c.269G>T G90V Exon 2		c.516+1G>A V144Gfs*85 IVS 4			c.965_966delTG V322Efs*92 Exon 8	c.1909A>C S637R Exon 11	
Patient	Proband	Proband	Son	Mother	Proband	Son	Proband	Brother	Proband
Age/sex	73/F	79/M	52/M	89/F	62/M	27/M	64/F	NA/M	62/M
Age at onset (years)	71	74	50	NA	60		63	NA	62
Onset symptom	Clumsiness of hands and unsteady gait	Dementia	Depression	NA	Slowness and gait disturabance	Asymptomatic	Unsteady gait	NA	Difficulty in driving a car
Parkinsonism	(+)	(+)	None	(+)	(+)	None	(+)	(+)	(+)
Levodopa responsiveness	(+)	NA		NA	(+)		(+)	NA	NA
Cognitive impairment	(+)	(+)	None	NA	None	None	Mild	NA	(+)
MMSE	16/30	13/30	30/30	NA	30/30	NA	24/30	NA	NA
HDS-R	NA	NA	NA	NA	NA	NA	22/30	NA	14/30
FAB	NA	3/18	NA	NA	NA	NA	Not examined	NA	NA
DAT SPECT	NA	Decreased	Normal	NA	Decreased	NA	Decreased	NA	NA
MIBG scintigraphy	NA	Decreased	Normal	NA	Decreased	NA	Decreased	NA	NA
Early H/M	NA	1.62	3.24	NA	1.43	NA	1.995	NA	NA
Delayed H/M	NA	NA	NA	NA	NA	NA	1.585	NA	NA
Autopsy	(+)	NA	NA	NA	NA	NA	(−)	NA	(+)
Lewy bodies	(+)								(+)
Reference	Yamada et al.^10^	Koyama et al.^14^		Koyama et al.^14^			This report	Kimura et al.^13^	

*F* female, *M* male, *NA* not applicable, *MMSE* Mini-Mental State Examination, *HDS-R* Hasegawa dementia scale revised, *FAB* frontal assessment battery, *DAT SPECT* dopamine transporter single photon emission CT, *MIBG scintigraphy* ^123^I-meta-iodobenzylguanidine myocardial scintigraphy

## Data Availability

The relevant data from this Data Report are hosted at the Human Genome Variation Database at 10.6084/m9.figshare.hgv.2603

## References

[1] Tadic V (2015). Primary familial brain calcification with known gene mutations: a systematic review and challenges of phenotypic characterization. JAMA Neurol..

[2] Wang C (2012). Mutations in SLC20A2 link familial idiopathic basal ganglia calcification with phosphate homeostasis. Nat. Genet.

[3] Keller A (2013). Mutations in the gene encoding PDGF-B cause brain calcifications in humans and mice. Nat. Genet.

[4] Nicolas G (2013). Mutation of the PDGFRB gene as a cause of idiopathic basal ganglia calcification. Neurology.

[5] Legati A (2015). Mutations in XPR1 cause primary familial brain calcification associated with altered phosphate export. Nat. Genet.

[6] Yao XP (2018). Biallelic mutations in MYORG cause autosomal recessive primary familial brain calcification. Neuron.

[7] Arkadir D (2019). MYORG is associated with recessive primary familial brain calcification. Ann. Clin. Transl. Neurol..

[8] Hsu SC (2013). Mutations in SLC20A2 are a major cause of familial idiopathic basal ganglia calcification. Neurogenetics.

[9] Lemos RR (2015). Update and mutational analysis of SLC20A2: a major cause of primary familial brain calcification. Hum. Mutat..

[10] Yamada M (2014). Evaluation of SLC20A2 mutations that cause idiopathic basal ganglia calcification in Japan. Neurology.

[11] Ding Y, Dong HQ (2018). A novel SLC20A2 mutation associated with familial idiopathic basal ganglia calcification and analysis of the genotype-phenotype association in Chinese patients. Chin. Med J. (Engl.).

[12] Nicolas G (2013). Phenotypic spectrum of probable and genetically-confirmed idiopathic basal ganglia calcification. Brain.

[13] Kimura T (2016). Familial idiopathic basal ganglia calcification: Histopathologic features of an autopsied patient with an SLC20A2 mutation. Neuropathology.

[14] Koyama S (2017). Clinical and radiological diversity in genetically confirmed primary familial brain calcification. Sci. Rep..

[15] Paschali A (2009). Dopamine transporter SPECT/CT and perfusion brain SPECT imaging in idiopathic basal ganglia calcinosis. Clin. Nucl. Med.

[16] Saito T (2010). Neuroradiologic evidence of pre-synaptic and post-synaptic nigrostriatal dopaminergic dysfunction in idiopathic Basal Ganglia calcification: a case report. J. Neuroimaging.

[17] Paghera B, Caobelli F, Giubbini R (2013). 123I-ioflupane SPECT in Fahr disease. J. Neuroimaging.

[18] Orimo S, Suzuki M, Inaba A, Mizusawa H (2012). 123I-MIBG myocardial scintigraphy for differentiating Parkinson’s disease from other neurodegenerative parkinsonism: a systematic review and meta-analysis. Park. Relat. Disord..

[19] Yamawaki M, Kusumi M, Kowa H, Nakashima K (2009). Changes in prevalence and incidence of Parkinson’s disease in Japan during a quarter of a century. Neuroepidemiology.

